# Will buffer zones around schools in agricultural areas be adequate to protect children from the potential adverse effects of pesticide exposure?

**DOI:** 10.1371/journal.pbio.2004741

**Published:** 2017-12-21

**Authors:** Robert B. Gunier, Asa Bradman, Kim G. Harley, Brenda Eskenazi

**Affiliations:** Center for Environmental Research and Children’s Health (CERCH), School of Public Health, University of California, Berkeley, Berkeley, California, United States of America; National Institute of Environmental Health Sciences, United States of America

## Abstract

California has proposed limiting agricultural pesticide use within 0.4 km of schools and childcare facilities. However, the 0.4-km buffer may not be appropriate for all pesticides because of differing toxicities, fate, and application methods. Living near pesticide use has been associated with poorer birth outcomes, neurodevelopment, and respiratory function in children. More research about exposures in schools, childcare facilities, and homes is needed. Despite incomplete science, this regulation is an important step to reduce potential exposures to children. The most vulnerable exposure period may be in utero, and future regulations should also aim to reduce exposures to pregnant women.

This Perspective is part of the *Challenges in Environmental Health: Closing the Gap between Evidence and Regulations Collection*.

The California Department of Pesticide Regulation (CDPR) recently proposed regulations limiting agricultural pesticide applications near school and childcare centers [1]. The new rules would prohibit pesticide applications using aircraft, air blast sprayers, chemigation, dust or powder, and fumigants made to produce an agricultural commodity within 0.25 miles (approximately 0.4 km) of a school or daycare on Monday through Friday from 6:00 AM to 6:00 PM. Most other agricultural pesticide applications would be prohibited within 25 feet (7.6 m) of school sites during the same time period. The proposed regulation addresses potential acute (short-term) pesticide exposures to children with the intent of providing an extra margin of safety for pesticide drift, which is defined as any off-target movement of pesticides from the treated area. These proposed regulations are largely based on emerging evidence from epidemiological studies, including ours (see below), that suggest that pesticide exposure may impact children’s health. In California, an estimated 2.5 million agricultural acres are within 0.33 miles (0.53 km) of an urbanized area [2]. Urbanization can create residential–farm conflicts, sometimes referred to as the “urban–agriculture edge” problem, in which neighbors can have problems with odor, dust, and pesticide spray drift [3]. In addition, a report from the California Department of Public Health summarized agricultural pesticide use within 0.4 km of public schools in California and found that some schools had high use of pesticides of public health concern applied nearby and that Hispanic children were more likely to attend schools near the highest use of pesticides, thereby raising important concerns about environmental justice [4]. Below, we outline the scientific basis for the CDPR’s policy decision of buffers around schools and the limitations of the data available.

Since 1999, we have conducted the Center for the Health Assessment of Mothers and Children of Salinas (CHAMACOS) Study, a partnership with the Salinas Valley community to examine the potential impact of pesticides and other environmental exposures on the health of pregnant women and children living in this agricultural community on the central coast of California. More than 600 children are currently enrolled in the study—half of whom have been followed since before birth and half since they were 9 years old—to assess their growth, health, and development. The CHAMACOS children are currently 15 to 17 years of age. The goal of our work is to understand the etiology of environmentally related disease and support policies to improve public health.

In the CHAMACOS study, we have observed associations between higher levels of biomarkers of pesticide exposure and poorer health and development. Specifically, higher concentrations of organophosphate pesticide (OP) urinary metabolites in maternal urine during pregnancy were associated with shortened gestational duration [5], greater odds of abnormal neonatal reflexes [6], pervasive developmental disorder and poorer mental development at 2 years of age [7], poorer attention and hyperactive behaviors at 5 years [8], and lower IQ at 7 years [9]. We are currently examining neurodevelopmental outcomes at older ages. We have also reported that both prenatal and child OP metabolite levels were associated with more asthma-related symptoms, and higher levels of OP pesticide metabolites in the urine of children between birth and 5 years of age were associated with reduced lung function at 7 years of age [10,11].

One major limitation of studies on the health effects of pesticides with short biological half-lives, such as OPs, is the assessment of exposure. In the Salinas Valley, more than 225,000 kg per year of OP pesticides were used during the pregnancy period of our study (1999 to 2000), but many other classes of pesticides were also used. Because several highly toxic OPs lack pesticide-specific biomarkers, we have used class-specific measurements of dialkylphosphate (DAP) urinary metabolites as an overall indicator of exposure [12]. However, limitations of DAP measurements include the following: (1) they do not reflect exposure to several OPs that do not devolve to DAPs, (2) it is not possible to determine whether they reflect exposure to more- or less-toxic OP pesticides, (3) they have a short half-life in the body and reflect only recent exposures [13], and (4) they may overestimate true OP exposure because they can reflect exposure to pre-formed DAPs in the environment and food as well as parent OP compounds [14,15]. These limitations may result in measurement error and bias towards the null hypothesis in health outcome studies [16]. Because of these limitations, it is especially important to confirm findings of an exposure response in multiple populations. For other classes of pesticides, such as fumigants, there are no valid biomarkers available to measure environmental exposures. Furthermore, biomarkers are not able to identify the source of exposure (i.e., diet, nondietary ingestion, or inhalation) and often do not provide guidance for policies to reduce exposures.

The CDPR has collected Pesticide Use Report (PUR) data for all agricultural pesticide applications in the state since 1990 [17]. The PUR data include the active ingredient applied, amount applied, formulation, application method, crop treated, acres treated, and location of the application. With more than 25 years of comprehensive agricultural pesticide use information, the PUR data provide a unique resource for evaluating associations between agricultural pesticide use and human health outcomes. In the CHAMACOS study, we have evaluated residential proximity to agricultural pesticide applications and children’s health and development utilizing PUR data and residential history information. We observed a relationship between higher use of the fumigant methyl bromide within 5 to 8 km of maternal residences during pregnancy and lower birth weight in their children [18]. We have also found that lower Full-Scale Intelligence Quotient (FSIQ) scores in children at 7 years of age were associated with (1) higher use of OP and other insecticides within 1 km of maternal residences during pregnancy [19], (2) higher use of the fumigants methyl bromide and chloropicrin within 8 km of children’s residences from birth to 7 years of age [20], and (3) higher agricultural use of certain mixtures of neurotoxic pesticides [21]. We found that the decrease in FSIQ associated with higher use of OP and carbamate insecticides within 1 km of maternal residences during pregnancy persisted in children at 10.5 years of age [22]. We also observed higher odds of respiratory symptoms and reduced lung function in children with higher use of sulfur, a low toxicity pesticide but known respiratory irritant, within 1 km of their residences during the year prior to pulmonary evaluation [23].

Several epidemiologic studies conducted in California have also used PUR data and found that higher nearby agricultural pesticide use was associated with poorer health outcomes of children. For example, children of mothers living within 0.5 km of higher agricultural use of the organochlorine pesticides dicofol and endosulfan during pregnancy had increased odds of developing autism [24], while in another study, greater odds of autism were seen among children whose mothers lived within 1.5 km of any agricultural use of OP or pyrethroid pesticides during pregnancy [25]. Studies using PUR data to evaluate the risk of birth defects related to agricultural pesticide use have found mixed results, with positive associations observed between the use of 2 carbamates (benomyl and methomyl) within 1 km of residences during pregnancy and neural tube defects in children [26]. Other studies have found few significant associations between agricultural pesticide use within 0.5 km and birth defects [27,28]. Finally, studies of childhood cancer have observed associations between leukemia and agricultural use of metam sodium and dicofol within 0.8 km of maternal residences during pregnancy [29].

There have been only a few studies that have evaluated the relationship of pesticide applications in agricultural communities and pesticide concentrations measured in outdoor air or in house dust. These studies have found correlations for some pesticides, but the strength of the correlation varies by pesticide and by distance over a fairly wide range, i.e., 4 to 8 km [30–35]. For example, outdoor air samples from homes located more than 0.25 km from fruit orchards had significantly lower concentrations of 2 OP pesticides (chlorpyrifos and azinphos-methyl) than did samples from homes within 0.25 km of orchards [36]. An air dispersion model developed for the fumigant 1,3-dichloropropene agreed well with available air monitoring data and estimated a 10-fold reduction in air concentrations at 0.4 km downwind of an application site and an additional 10-fold reduction in air concentrations from 0.4 km to 1 km [31]. A recent study using published data on pesticide concentrations in dust found that the average concentrations were 64% lower in homes located 0.25 km compared with 0.02 km from treated fields [37]. From CHAMACOS and other studies, we have observed that nearby agricultural use of only some pesticides is correlated with levels of pesticides in residential house dust samples, and these correlations range from weak to moderate [38,39].

Overall, we have limited data on air and dust concentrations in relation to nearby pesticide applications and on the fate and transport of specific pesticides. For certain common pesticides, such as neonicotinoids and glyphosate, we have no information on the relationship of PUR data and environmental or biological measures. In addition, estimating human exposure from PUR data would likely underestimate exposure because it considers only residential exposure and not the individual’s cumulative exposure across multiple settings, such as at childcare facilities, schools, or work in addition to residential exposure. Estimating pesticide exposure from PUR data could be improved by considering local meteorological conditions, the physical characteristic of the pesticide (e.g., persistence, volatility etc.), and method of pesticide application (e.g., aerial versus ground).

Therefore, identifying the correct buffer or distance from treated fields that would be sufficient to protect public health is complex. Given the different physical properties of pesticides, it is unlikely that a single distance would be adequate to address the health risks of all pesticide exposure, including those less studied [40,41]. Also, buffers might differ by the pesticide mixtures being applied because together they may augment health effects [21]. Determination of the proper buffer should be based on the published literature of pesticide exposure and health effects, but there may be more sensitive health endpoints not yet studied that would require a smaller buffer to protect the public’s health. Although the scientific data necessary to determine the ideal distance or buffer to reduce pesticide exposure and adverse health effects are inadequate, policies to protect children are warranted given the evidence that pesticide exposure may increase risks to children’s health. However, we note that implementing buffers around schools considers potential exposure to children but does not aim to reduce exposure to pregnant women in the general community, and exposure during the in utero period may have the greatest adverse effects.

In addition to requiring a buffer, policies to establish other protective measures to reduce exposure to pregnant women as well as children should be taken. Other potential solutions include good neighbor actions by agricultural operators such as notifying nearby residents of planned applications and protective city planning and zoning for new residential developments [3], including a gradual conversion of fields near schools and residential areas to organic—although the use of some organically approved pesticides, such as sulfur, also need to be carefully monitored. The ideal strategy for protecting children and pregnant women is an overall reduction in the use of agricultural pesticides. This process has already begun. Since we began the CHAMACOS study in 2000, overall insecticide use in California has declined, and OP pesticide use in California has declined by 60% from approximately 2.9 million to approximately 1.1 million kg per year in 2015 (Fig 1). However, the use of some classes of pesticides is increasing, as illustrated by the almost 9-fold increase in neonicotinoid pesticide use from approximately 45,500 kg in 2000 to approximately 395,500 kg in 2015 (Fig 1), and overall pesticide use has changed very little since 2000, with approximately 91 million kg applied per year [17].

**Fig 1:**
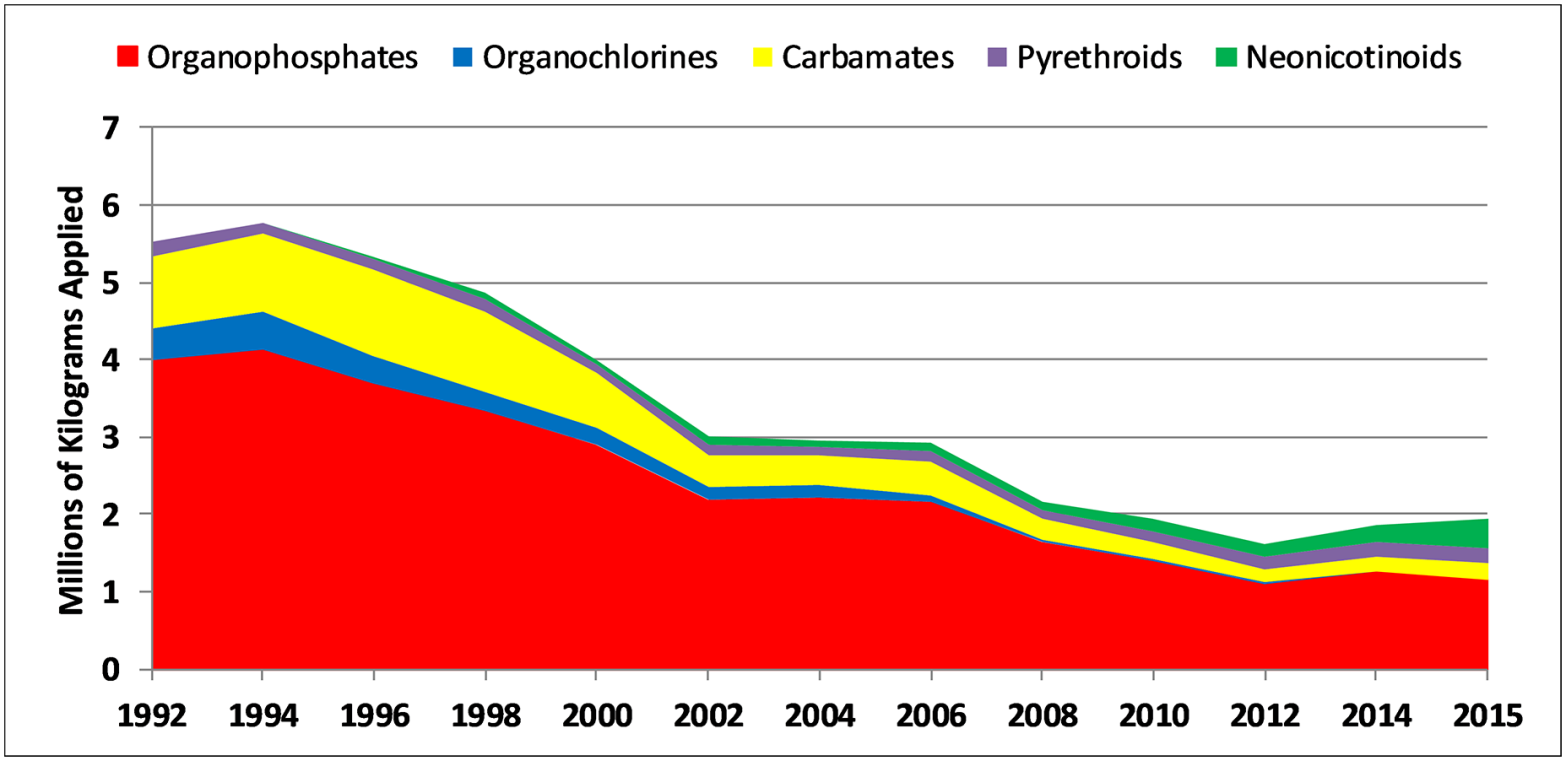
Trends in agricultural insecticide use in California from 1992 to 2015.

We have learned over the last 2 decades that agricultural pest control is complicated, requires flexibility, and needs a diverse set of pest control options to successfully produce the food that is so vital to California’s population and economy. Solutions require thoughtful approaches. CDPR is a leader in this area, as noted in the 2015 National Academy of Sciences review of the CDPR pesticide risk assessment procedures [42]. CDPR has instituted important programs to support Integrated Pest Management and to use fewer pesticides more safely and efficiently. Integrated Pest Management is a method of reducing economic, human health, and environmental risks from pests and pest management strategies [43]. We encourage continued promotion of these practices to minimize agricultural pesticide use and to develop less toxic alternatives. We commend CDPR’s “reduced risk” pest management philosophy, which has led to these changes in application methods and substantially lowered the use of OP insecticides in California during the last decade and has engaged community and stakeholders in the dialogue to reduce exposures to children in schools. Future studies of pesticide exposure and human health effects could be designed by researchers in collaboration with CDPR to better characterize appropriate buffer distances from treated fields that would be protective of children’s health. We anticipate that CDPR will continue to work with researchers, farmers, and residents in agricultural communities to protect human health and the environment. Despite limitations, we believe that the proposed regulation to implement buffers is a step in the right direction for preventing potential pesticide exposure to children in agricultural communities, and we therefore support the use of buffer zones around schools and daycares to reduce children’s exposure during the day as an important first step.

## Abbreviations

CDPR: California Department of Pesticide Regulation
CHAMACOS: Center for the Health Assessment of Mothers and Children of Salinas
DAP: dialkylphosphate
FSIQ: Full-Scale Intelligence Quotient
OP: organophosphate pesticide
PUR: Pesticide Use Report
